# Severe Traumatic Brain Injury and Pulmonary Embolism: Risks, Prevention, Diagnosis and Management

**DOI:** 10.3390/jcm13154527

**Published:** 2024-08-02

**Authors:** Charikleia S. Vrettou, Effrosyni Dima, Nina Rafailia Karela, Ioanna Sigala, Stefanos Korfias

**Affiliations:** 1 First Department of Critical Care Medicine, Evangelismos Hospital, Medical School, National & Kapodistrian University of Athens, 10676 Athens, Greecerafaelakarel@gmail.com (N.R.K.);; 2 Department of Neurosurgery, Evaggelismos General Hospital of Athens, 10676 Athens, Greece

**Keywords:** traumatic brain injury, pulmonary embolism, intensive care unit

## Abstract

Severe traumatic brain injury (sTBI) is a silent epidemic, causing approximately 300,000 intensive care unit (ICU) admissions annually, with a 30% mortality rate. Despite worldwide efforts to optimize the management of patients and improve outcomes, the level of evidence for the treatment of these patients remains low. The concomitant occurrence of thromboembolic events, particularly pulmonary embolism (PE), remains a challenge for intensivists due to the risks of anticoagulation to the injured brain. We performed a literature review on sTBI and concomitant PE to identify and report the most recent advances on this topic. We searched PubMed and Scopus for papers published in the last five years that included the terms “pulmonary embolism” and “traumatic brain injury” in their title or abstract. Exclusion criteria were papers referring to children, non-sTBI populations, and post-acute care. Our search revealed 75 papers, of which 38 are included in this review. The main topics covered include the prevalence of and risk factors for pulmonary embolism, the challenges of timely diagnosis in the ICU, the timing of pharmacological prophylaxis, and the treatment of diagnosed PE.

## 1. Introduction

The management of severe traumatic brain injury (sTBI) in the intensive care unit (ICU) presents numerous challenges, while the level of evidence on many sTBI-related topics is still low compared to other aspects of critical care [1,2]. In addition to the risks associated with primary brain injury, sTBI patients also face risks related to critical illness and the limitations sTBI imposes on other aspects of intensive care management, including but not limited to the timely initiation of venous thromboembolism (VTE) prophylaxis [3]. Furthermore, the brain interacts with other body organs, and the occurrence of complications such as pulmonary embolism (PE) may lead to additional secondary brain injury [4]. The simultaneous occurrence of VTE events, particularly PE, poses a significant challenge to intensivists due to the risks associated with therapeutic anticoagulation in patients with brain injuries, most often related to the risk of hematoma expansion [1]. Managing cases of high-risk PE is particularly difficult because sTBI patients often have contraindications to thrombolysis. Additionally, the use of rescue therapies for respiratory failure, such as prone positioning or extracorporeal membrane oxygenation (ECMO), has not been extensively studied in this population, and their effects on the damaged brain are not well understood [5]. For ECMO use, in particular, there is limited experience primarily because its application requires anticoagulation, which exposes the injured brain to the risk of hematoma expansion [6,7].

There are numerous pathophysiologic considerations relevant to thrombotic risks that intensivists need to keep in mind, even before ICU admission, when the patient is still assessed in the out-of-hospital or emergency room (ER) environment. Immediately after trauma, a coagulopathic state often occurs. This state is marked by hyperfibrinolysis, endothelial dysfunction, and platelet consumption and dysfunction [8]. Management at this stage primarily focuses on stopping any life-threatening bleeding, typically from extracranial sources. The next immediate priority is addressing coagulopathy, which is notoriously aggravated by acidosis and hypothermia. Current evidence suggests that tranexamic acid (TXA) administration is beneficial to all bleeding polytrauma patients within the first three hours from injury. TXA prevents the breakdown of fibrin, which helps reduce bleeding. However, in some cases, its use may increase the risk of thrombosis. The CRASH-3 trial showed that while TXA reduced TBI-related deaths in patients with mild-to-moderate TBI, it was not so beneficial in cases of isolated severe traumatic brain injury [9]. Although general treatment strategies for coagulopathy in sTBI patients are similar to those for patients with extracranial injuries, some experts recommend maintaining a higher platelet count in the former patient group and suggest more than 100.000 per microliter of blood [8].

After the first “golden” hours [10] and later in the clinical course of sTBI, the risk of venous thromboembolism (VTE) is increased due to hypercoagulability. This is driven by excessive thrombin generation and inflammation and further aggravated by immobilization [11]. This problem is ideally addressed by administering pharmaceutical VTE prophylaxis, such as low-molecular-weight heparin (LMWH), which, however, may carry the risk of intracranial lesion expansion. Therefore, even before starting LMWH, intermittent pneumatic compression is an option for VTE prophylaxis. After 24–72 h, once hemostasis is achieved and repeated neuroimaging shows no hematoma progression, LMWH can likely be initiated without an increased risk of hemorrhage. Considering the numerous factors clinicians must evaluate before initiating anticoagulation, it is not surprising that the timing of LMWH initiation varies significantly across different centers and studies, ranging from one to seven days [12].

After an sTBI patient is admitted to the ICU, the clinical suspicion and definitive diagnosis of PE can be challenging. Regarding clinical suspicion, cardinal manifestations of PE—such as hypoxia, asynchrony with mechanical ventilation, tachycardia, and hemodynamic instability—may, in the ICU setting, be attributed to other conditions, such as pneumonia, sepsis, catecholamine-induced cardiomyopathy, or the trauma itself [4]. Regarding diagnosis, the use of chest and heart ultrasound may be limited by concomitant chest trauma [13]. Additionally, coexisting pulmonary disease can obscure the causes of right ventricular dysfunction or acute pulmonary hypertension [14]. Regarding blood biomarkers for PE diagnosis and risk stratification, such as cardiac troponins and natriuretic peptides [15], these may also be influenced by heart dysfunction, such as cardiac injury, fluid overload, and neurogenic pulmonary edema [4]. Finally, continuous hemodynamic monitoring trends may also be affected by the aforementioned conditions. As a result, when PE is suspected, intensivists mostly rely on CT pulmonary angiogram, which is the gold standard for diagnosis [14] but may also pose logistic difficulties in critically ill, unstable patients [16].

Evidence on the best management practices for diagnosed PE in sTBI is also limited, necessitating an individualized approach where the pros and cons of each option are carefully balanced [17,18]. While therapeutic anticoagulation (AC) is an option for low- and medium-risk patients, it may need to be delayed due to the increased risk of hematoma expansion, making the timing of PE occurrence an important parameter that affects clinical decisions. Inferior vena cava filters (IVCFs) are a consideration when the bleeding risk is high, but they come with their contraindications and risks [14,19,20,21].

The most challenging scenario is managing sTBI alongside high-risk PE. Severe high-risk acute PE is characterized by the occurrence of cardiac arrest, obstructive shock, or persistent hypotension, with systolic blood pressure < 90 mmHg, or a drop in blood pressure by more than 40 mmHg for over 15 min, not attributable to other causes [22]. For severe, high-risk PE, current guidelines advise thrombolysis, but this is contraindicated if the patient has a recent sTBI. If thrombolysis is contraindicated, percutaneous catheter-directed treatment should be considered, despite its risk of bleeding. However, percutaneous catheter-directed treatment may not be available in many hospitals, except for level 1 trauma centers [22]. Further respiratory and circulatory support, such as extracorporeal membrane oxygenation (ECMO), may have to be considered to support the patient. The need for concomitant anticoagulation remains a concern with these strategies. However, if they are available, they should be considered carefully and not excluded on the sole basis of concomitant sTBI. Recent case series with the successful implementation of both VV- and VA-ECMO in sTBI patients show promising results [5,7,17].

In recent times, an increasing number of elderly and frail patients have been admitted with TBI [23], and the special aspects of their care are reported [24]. These patients differ from other critically ill populations because they have high comorbidity rates and even higher risks of thrombosis. In addition, many of them may be on anticoagulants for VTE or other reasons such as atrial fibrillation, prosthetic valves, or heart disease, before ICU admission. The management of PE in the elderly with TBI needs to take into consideration the special needs of this population group, quality of life expectations, comorbidities, and pharmacokinetic changes that come with age [25].

In light of the above, we decided to conduct a literature review to explore the medical literature and advancements regarding PE in sTBI populations. We aimed to include the new clinical research published after the latest ESC guidelines for the management of PE in 2019.

## 2. Materials and Methods

**Figure 1:**
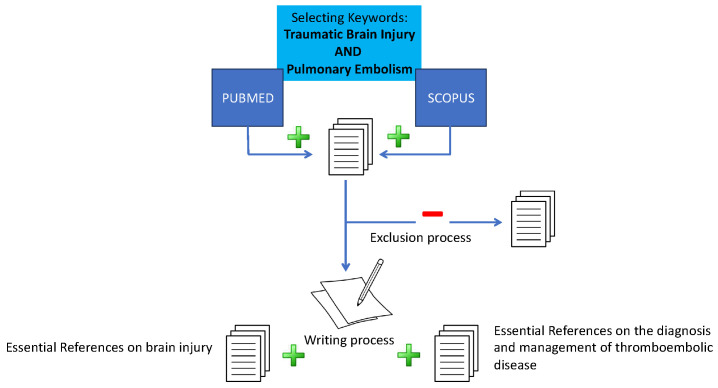
Flowchart of the search procedure. After the initial search and paper selection, and during the writing process, additional publications were included, such as guidelines and papers pertinent to the covered topics. The green signs indicate the steps in which papers were added, while the red sign indicates the step in which publications were excluded.

We conducted a literature review on sTBI and PE to identify the recent English-language publications on this topic from the past five years. Specifically, we searched PubMed and Scopus for papers published in the last five years containing the terms “pulmonary embolism” and “traumatic brain injury” in their titles or abstracts. We excluded publications referring to children, other non-thrombotic types of embolism such as fthose caused by at or a foreign body, and anticoagulation management for other clinical entities such as prosthetic valves or atrial fibrillation. Additionally, papers discussing anticoagulation reversal were excluded. Two independent authors selected the papers, resolving any discrepancies in the initial selection by consensus. During the writing and revision process, additional relevant papers were incorporated by the authors, including guidelines and publications pertinent to the specific topics discussed in each section. Figure 1 presents the flowchart of the search for this review.

## 3. Results

**Figure 2:**
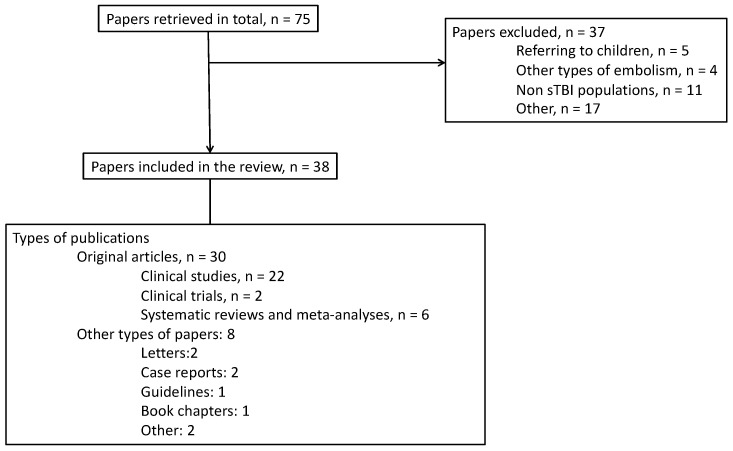
Flowchart of the search results for this review.

Our search revealed a total of 75 papers. After excluding 37 papers, 38 publications were included in our review. Among these, 30 were original publications, 2 were case reports, and 2 were letters to the editor or editorials. Figure 2 presents the search flow chart, while Table 1 summarizes the original studies and their main findings that were relevant to the topic of this review.

Among the 30 original publications, 22 were observational studies, 2 were clinical trials, and 6 were systematic reviews or meta-analyses. Most of the 22 observational studies were single-center retrospective studies. The two randomized controlled trials focused on the effects of tranexamic acid (TXA), as did most of the meta-analyses. Other relevant topics in our review included risk factors for PE, timing, and dosage of prophylactic and therapeutic anticoagulation, and the use of IVCFs (Table 1). Additional publications covered topics such as the use of ECMO in fulminant PE and sTBI [17] and brain–lung interactions in sTBI [26].

**Table 1:** Summary of original publications on severe traumatic brain injury and pulmonary embolism.

Authors	Methods	No of Patients	Topic
Song et al., 2024 [27]	SR + MA		TXA does not affect the incidence of PE.
Zhang et al., 2024 [28]	MA		TXA does not affect the incidence of PE.
Cole et al., 2024 [29]	Single center, retrospective	818	Risk factors for PE
Condon et al., 2024 [30]	Multiple center, retrospective	14,926	LMWH was associated with lower mortality risk compared to UH in geriatric patients.
Park et al., 2023 [31]	Single center, prospective	120	Crcl predicts goal enoxaparin dose
Jakob et al., 2023 [32]	Multiple center, retrospective	75,570	Risk factors for PE
Wu et al., 2023 [33]	Secondary analysis of PCD	881	Early vs. late initiation of VTE prophylaxis
Chen et al., 2023 [34]	Multiple center, retrospective	847	Risk factors for PE
Samuel et al., 2023 [35]	Single center, retrospective	103	Timing of therapeutic AC in TBI with PE
The PATCH-Trauma Investigators and the ANZICS Clinical Trials Group 2023 [36]	Multiple center RCT	1310	TXA effect
Zheng et al., 2022 [37]	SR + MA		EPO in TBI
Hazelton et al., 2022 [38]	Multicenter prospective observational study	1623	WB resuscitation in TBI
Ali et al.,2022 [39]	Multiple center, retrospective	2754	Risk of PE in THC+ cases
Ali Basil Ali et al., 2022 [40]	Multiple center, retrospective	37,988	Risk of PE in DC vs. CO
Perissier et al., 2022 [41]	Single center, prospective	120	Reasons for late initiation of VTE prophylaxis
Fletcher-Sandersjöö et al., 2022 [42]	Retrospective observational	848	Clinical significance of VTE in TBI
Byrne et al., 2022 [43]	Multiple center, retrospective	4951	Early vs. late initiation of VTE prophylaxis
Rivas et al., 2022 [44]	Multiple center, retrospective	264	Early vs. late initiation of VTE prophylaxis
El-Menyar et al., 2022 [45]	Multiple center RCT	220	Effect of second TXA dose
Galaher et al., 2021 [46]	Single center, retrospective	806	Diagnostic yield of TTE
Gates et al., 2021 [47]	Single center, prospective	1698	Titration of prophylactic AC to anti-Xa levels
Elkbuli et al., 2021 [48]	Single center, retrospective	413	Prophylactic IVC
Elkbuli et al., 2020 [49]	Single center, retrospective	513	Timing of prophylactic IVC placement
Chipman et al., 2020 [50]	Single center, retrospective	50	Timing of therapeutic AC in TBI with PE
Ahmed et al., 2020 [51]	Multiple center, retrospective	2370	Risk of PE in DC
Lu et al., 2020 [52]	SR + MA		Early vs. late initiation of VTE prophylaxis
Zhang et al., 2020 [53]	Single center, retrospective	144	Risk of pharmaceutical immobilization
July et al., 2020 [54]	MA		TXA effect
Chen et al., 2020 [55]	MA		TXA effect
Rabinstein et al., 2019 [56]	Single center, retrospective	77	PICC as a risk factor

SR, systematic review; MA, meta-analysis; TXA, tranexamic acid; PE, pulmonary embolism; LMWH, low-molecular-weight heparin; UH, unfractionated heparin; Crcl, creatinine clearance; AC, anticoagulation; EPO, erythropoietin; RCT, randomized control trial; TBI, traumatic brain injury; WB, whole blood; THC, tetrahydrocannabinol; DC, decompressive craniectomy; CO, craniotomy; VTE, thromboembolic prophylaxis; TTE, transthoracic echo; IVC, inferior vena cava; PICC, peripherally inserted central catheter.

### 3.1. Incidence of PE in sTBI

The incidence of PE varied in different studies reporting results from sTBI populations, but the rates according to the recent literature are low, probably also due to the increased application of VTE prophylactic measures [57,58]. In a retrospective study, the overall incidence of VTE was 9.1%, with 3.2% being cases of PE. The median time to diagnosis for PE was five days [29,59]. In another recent study, the incidence of PE in the entire population was 6.9%, with most PE cases occurring in the subpopulation with additional injuries. The risk factors for VTE in polytrauma patients with sTBI were delayed initiation of prophylactic anticoagulant therapy and mechanical thromboprophylaxis [34].

### 3.2. Risk Factors for PE in sTBI

We specifically searched for any new evidence on VTE risk factors particularly referring to sTBI patients, keeping in mind that there are scoring systems available in the literature for assessing VTE risk [60]. In a retrospective observational study, investigating the incidence, risk factors, and clinical significance of thromboembolic events in patients with moderate-to-severe TBI, independent predictors of VTE events included the length of ICU stay, increased body weight, and the presence of a skull fracture. However, VTE events did not significantly impact the 12-month outcomes, even after adjusting for potential confounders through propensity score matching [42]. Furthermore, a retrospective study of over 70,000 patients found a trend for penetrating trauma linked to a higher risk of VTE and increased mortality from PE [32].

Some studies investigated whether craniotomy (CO) and decompressive craniectomy (DC) may influence the risk of pulmonary complications, including PE [40,51]. Using a propensity-score-matched analysis, Ahmed et al. found no significant difference in PE incidence between patient groups undergoing these procedures [51]. In contrast, another study reported an association between DC and VTE risk [40]. Post-DC VTE was linked to poorer outcomes, longer hospital stays, and higher hospitalization costs. Identified risk factors for post-DC thromboembolic events included older age, obesity, electrolyte imbalance, chronic lung disease, spine injury, and concomitant infection [40].

The presence of a central femoral line is a previously identified risk factor for VTE [14,61,62]. In a recent retrospective analysis, femoral arterial catheters were more commonly used in critically ill patients, particularly those with shock or TBI. Their complication rate was lower than reported in other studies, with a 12% incidence of mechanical complications and 42% incidence of VTE, none of which were linked to confirmed PE [63]. On the contrary, peripherally inserted central venous catheters were associated with a higher incidence of upper extremity VTE. A study exploring the use of sequential compression devices on the arms to reduce central line-associated VTE was terminated early due to higher rates of VTE in the treatment arm [56].

The use of muscle relaxants and barbiturates for deep sedation has been correlated with a higher incidence of VTE among sTBI patients [43,64,65,66,67,68], but the urgency for intracranial pressure (ICP) control in sTBI patients can outweigh the increased thromboembolic risk. Enhanced surveillance and screening during prolonged sedation and immobilization are thereby recommended. A study by Zhang et al. indicated an elevated incidence of VTE in patients requiring pharmaceutical immobilization for ICP control, although the number of PE cases in this cohort was very low [53,59]. Finally, a study found that preinjury tetrahydrocannabinol use in TBI patients did not significantly affect in-hospital outcomes such as mortality, length of stay, and the rates of cardiac arrest, PE, DVT, or acute respiratory distress syndrome [39,69].

### 3.3. The Role of TXA

The use of TXA in sTBI management remains debated, and increased risk of PE with the use of TXA was reported in the past in sTBI patients [70]. Recently, the PATCH-Trauma Investigators and the ANZICS Clinical Trials Group reported prehospital administration of TXA followed by an 8 h infusion did not improve survival rates with a favorable functional outcome at 6 months compared to placebo, in patients with major trauma [36]. This result makes the possible additional VTE risks with the use of TXA more relevant for sTBI patients.

There are six recent systematic reviews and meta-analyses on TXA administration in sTBI patients. One review found that TXA was associated with reduced mortality and hemorrhagic expansion but did not affect the need for neurosurgical intervention and unfavorable Glasgow Outcome Scale (GOS) scores. Surprisingly, vascular occlusive events were slightly lower in the TXA group in a subgroup analysis of RCTs with a low risk of bias [54]. Chen et al. reported that TXA administration resulted in significantly reduced mortality and hemorrhagic mass growth but had no notable impact on neurosurgery, extracranial surgery, unfavorable outcomes, PE, or deep vein thrombosis (DVT) [55]. Rowel et al. concluded that out-of-hospital TXA administration within 2 h of injury, compared with placebo, did not significantly improve the 6-month GOS but also did not increase the risks of PE [71]. More recently, El-Menyar et al. found that a second in-hospital dose of TXA did not improve mortality rates, transfusion needs, thromboembolic complications, organ failure, or hospital length of stay compared to a single prehospital dose [45].

A meta-analysis incorporating ten RCTs reported that TXA administration in TBI patients resulted in a significant reduction in mortality and hemorrhage growth volume, with no significant impact on PE incidence [27]. Similarly, Zhang et al. reported no significant increase in the risks of adverse events and noted that early administration of TXA (within 3 h from injury) may significantly decrease the likelihood of intracranial hemorrhage growth in TBI patients [28]. Overall, the body of evidence produced during the last five years does not support an increased risk of VTE or PE related to TXA use.

### 3.4. The Role of Transfusions and Transfusion Thresholds

The effects of blood product transfusions and transfusion thresholds on sTBI outcomes were recently examined in an RCT, which reported no association between transfusion strategy and VTE events in adults with moderate-to-severe TBI and anemia [72]. Furthermore, a multicenter prospective study of trauma patients who received whole blood or blood components during resuscitation reported no differences in the rates of acute kidney injury, VTE/PE, or other pulmonary complications [38]. Erythropoietin (EPO) use has also been applied in an attempt to reduce the transfusion requirements in sTBI. Zheng et al. investigated the efficacy and safety of EPO regimens in TBI. The study found a trend between EPO dosage and reduced mortality but also increased VTE rates [37].

### 3.5. Timing of Prophylaxis Initiation

Despite existing guidelines, a recent prospective study showed delayed (>36 h) pharmacologic VTE prophylaxis initiation in the majority of trauma patients. Five independent factors were associated with this delay, among which was the estimated risk of neurological deterioration and planned surgery necessitating interruption of anticoagulation. Prescribing delays in anticoagulation were highlighted as an area for further improvement in sTBI care [41].

In a cohort study involving 4951 patients, delayed prophylaxis after a neurosurgical intervention was linked to a higher risk of thrombosis, whereas earlier initiation of prophylaxis increased the likelihood of needing a repeated neurosurgical procedure, implying that early pharmacologic prophylaxis after TBI reduces the risk of thromboembolic complications but raises the risk of subsequent neurosurgical interventions [73]. These findings are different from those of a systematic review and meta-analysis on early (≤72 h) vs. late (>72 h from admission) antithrombotic prophylaxis. In this meta-analysis, the authors did not find a statistically significant difference in the incidence of lesion progression or mortality between the early and the late groups, but in the early group, the incidence of VTE was significantly lower [52]. In a retrospective study conducted on adult TBI patients with blunt injury, early (≤24 h) versus late (>24 h) chemoprophylaxis initiation did not affect bleeding progression or VTE rates. The average time to VTE prophylaxis initiation was 17 h for the early group and 47 h for the late group [44]. In a secondary analysis of the prospective multicenter Consortium of Leaders in the Study of Thromboembolism study, early (≤48 h) initiation of pharmacologic VTE prophylaxis was associated with decreased VTE rates but not different PE rates, without increased risk of hematoma expansion. Additionally, enoxaparin was found to be superior to UFH for prophylaxis purposes in patients with sTBI [33].

### 3.6. Agents and Dosage of Pharmaceutical VTE Prophylaxis

LMWH has been linked to a reduced risk of VTE and mortality compared to UFH in sTBI, particularly in geriatric patients [30]. Enoxaparin has increased bioavailability, longer plasma half-life, and more predictable pharmacokinetics and pharmacodynamics compared with unfractionated heparin [32,74,75]. It interacts less with platelets, which may reduce bleeding complications compared with UFH, has a lower incidence of heparin-induced thrombocytopenia, and is not associated with the osteoporosis observed with heparin treatment [76]. Higher doses are now considered the standard of care [74]. A dosage of 40 mg twice daily is recommended as the standard for most trauma patients, as 30 mg twice daily often results in inadequate pharmacologic prophylaxis [74]. According to recent guidelines, patients with brain and spine trauma should be initiated on 30 mg of enoxaparin twice daily and considered for dose adjustment by anti-Xa level (anti-Xa peak level goal of 0.2 to 0.4 IU/mL, anti-Xa trough levels goal > 0.1 IU/mL) [75,77]. A recent study showed a statistically significant reduction in VTE rates (but not a reduction in PE rates) with this strategy. Implementing this type of protocol requires diligence from both physicians and pharmacists [47]. It has been suggested that creatinine clearance (CrCl) is a more accurate predictor for determining high and low enoxaparin dose requirements than weight alone. A retrospective review at an urban, academic Level I trauma center supported this hypothesis, indicating that CrCl better predicts the appropriate enoxaparin dose for TBI patients compared with weight-based dosing [31]. Thromboelastography (TEG) has not been validated for monitoring pharmacologic prophylaxis. TEG with platelet mapping could assist in platelet function assessment and guidance for the addition of aspirin to the pharmacologic prophylaxis regimen in selected cases [31].

### 3.7. The Role of IVCFs

Recently, two retrospective studies on IVCFs in sTBI patients were published. One evaluated the outcomes in severely injured patients who received IVCFs. For patients with critical head injuries (AIS-Head ≥ 3), IVCF placement was associated with lower in-hospital mortality compared to VTE chemoprophylaxis [48]. Another single-center retrospective review examined adult trauma patients who underwent prophylactic IVCF placement, dividing them into two groups based on the time to filter: within 0–48 h and after 48 h. The authors report that early IVCF placement was associated with shorter ICU and hospital stays [49].

### 3.8. Timely PE Suspicion and Diagnosis

The use of routine duplex ultrasonography to detect asymptomatic DVT varies widely among trauma centers. While screening for DVT with lower-extremity ultrasonography increases detection rates, it does not correlate with reduced PE occurrence. Therefore, routine VTE screening is not recommended for all trauma patients. However, routine screening for DVT in high-risk asymptomatic trauma patients (risk assessment profile > 10, see also Table 2) [78,79] may enhance VTE identification and lower the rate of symptomatic PE. Thus, routine lower extremity duplex screening is advisable only for asymptomatic patients at high risk for VTE [60]. Transthoracic echocardiography (TTE) is frequently employed for assessing critically ill surgical patients, yet most examinations are normal and do not lead to changes in clinical management. A recent retrospective study reviewed TTE results from patients in a tertiary trauma/surgical ICU over 2.5 years, revealing that 3% of exams identified a previously undiagnosed PE [46]. This evidence is not against the immediate implementation of TTE or transesophageal echo when there is suspicion of high-risk PE [14].

### 3.9. Treatment of PE in TBI

The timing of anticoagulation initiation for VTE management in sTBI also varies significantly. A retrospective study evaluated patients with traumatic or vascular brain injuries and DVT or PE. It aimed to compare clinical outcomes, measured by the modified Rankin Score (mRS), in patients who began therapeutic anticoagulation early (≤3 days) versus late (>3 days) after VTE diagnosis. The study excluded patients on anticoagulation before admission, those diagnosed with VTE on admission, or those with non-brain injuries. The outcomes were similar between the early and late anticoagulation groups. There was a trend toward better outcomes in the early group, particularly in those diagnosed with a VTE between 4 and 7 days. Only older age was significantly associated with worse outcomes [35]. Another retrospective single-center study found that initiating therapeutic anticoagulation early (<7 days from injury) was not linked to worse outcomes. The study concluded that intracranial hemorrhage should not preclude early anticoagulation treatment for PE [50].

IVCFs may be considered in the setting of proximal DVT or PE when there is a contraindication to appropriate therapeutic anticoagulation. While consensus guidelines provide conflicting recommendations and most studies have been observational, among patients diagnosed with an acute proximal DVT or PE who cannot receive adequate therapeutic anticoagulation, an IVCF should be considered to reduce the rate of recurrent PE without altering the mortality rate [74].

### 3.10. Management of High-Risk PE in sTBI

The initial approach to managing hemodynamic instability in patients with high-risk PE depends on the preload status of the right ventricle (RV). For patients without signs of increased right-sided preload (central venous pressures < 15 mmHg), the goal is to raise RV preload using intravenous fluids. For those with already elevated preload, the focus shifts to enhancing RV function with inotropes and vasopressors, such as norepinephrine, epinephrine, and vasopressin. These medications may also be necessary to boost cardiac output or address hemodynamic instability associated with significant tachycardia [14,80,81].

In cases of RV failure and obstructive shock, veno-arterial ECMO (VA-ECMO) can offer circulatory support and serve as a rescue therapy for patients unresponsive to standard treatments or those with contraindications to thrombolysis. However, ECMO carries a significant bleeding risk, necessitating a careful anticoagulation strategy. No randomized controlled trials have evaluated the efficacy and safety of ECMO in high-risk PE. While some small observational studies have suggested potential benefits of using ECMO (in combination with anticoagulation) without reperfusion, others have not [80,81,82,83,84,85]. Current guidelines recommend considering VA-ECMO only in combination with alternative reperfusion strategies for patients with PE and refractory circulatory collapse or cardiac arrest [14,18,80].

In cases of high-risk PE, immediate referral for reperfusion treatment is essential based on clinical assessment. Testing for laboratory biomarkers such as cardiac troponins or natriuretic peptides is not necessary for immediate therapeutic decisions. When thrombolytic therapy is contraindicated, such as in cases of concomitant sTBI, options are limited to percutaneous mechanical-catheter-directed thrombectomy/embolectomy and surgical embolectomy. Catheter-based approaches have gained considerable interest over the past decade considering the complexity and risks associated with surgical embolectomy. Current guidelines indicate that mechanical-catheter-directed thrombectomy does not offer significant advantages over thrombolysis for most patients with a high-risk PE [14]. However, for patients with brain injuries, systemic thrombolysis is contraindicated and catheter-directed thrombolysis still exposes the injured brain to the thrombolytic agent in the circulation [86,87,88,89]. In such cases, interventional reperfusion with percutaneous mechanical-catheter-directed thrombectomy presents a reasonable option. Nonetheless, while these techniques are increasingly applied, there is limited evidence for patients with brain injuries [90,91,92,93]. Surgical embolectomy is another option and has been applied in resource-limited settings [14,94]. However, brain damage from prolonged hypoxemia and hypotension before surgery, as well as brain damage due to the long period of circulatory arrest required for the intervention, remain significant concerns limiting the application of this strategy in sTBI.

## 4. Discussion

The incidence of PE, particularly high-risk PE, in sTBI populations varies between studies and is challenging to determine, but overall, the reported PE rates are lower than 6% [29,34,59]. Recent evidence indicates that risk factors for VTE and PE in sTBI populations include the presence of skull fractures, increased body mass index, penetrating head trauma, decompressive craniectomy, and prolonged sedation and immobilization for intracranial hypertension management [28,32,39,40,42,51]. The use of TXA was not associated with an increased risk of VTE, but it also did not improve outcomes. However, new evidence suggests that early TXA administration can reduce hemorrhagic expansion. It is reasonable to conclude that the use of TXA can be considered and individualized according to the estimated benefits and risks for each patient [28,45,55,71]. Overall, blood transfusions were previously known to increase VTE/PE risk. More recent evidence does not support the use of EPO in sTBI to reduce transfusion requirements [61,62].

According to previously known evidence, mechanical prophylaxis implementation is encouraged as soon as possible for moderate-to-high VTE-risk patients [74]. In contrast, the initiation timing of pharmacologic prophylaxis in sTBI should be personalized, considering various factors, including the severity of the injury. Notably, the progression of TBI occurs in about 10% of patients with a stable follow-up CT, regardless of whether pharmacologic prophylaxis with LMWH is provided or not [74,95]. The American Society of Hematology 2019 guidelines state that for patients at high risk of VTE, it is advised to add mechanical prophylaxis (preferably intermittent pneumatic compression) to pharmacological prophylaxis if there is no contraindication due to lower extremity injury. The guidelines further recommend against using an IVCF for primary VTE prevention and against routine surveillance with venous compression ultrasound [96].

Clinical practice and research acknowledge that TBI is a heterogeneous disease in which increased severity of intracranial hemorrhage is also associated with higher rates of hematoma progression. To optimize VTE prophylaxis, the American Association for the Surgery of Trauma and the American College of Surgeons-Committee on Trauma collaborated to develop consensus recommendations, according to which the primary determinant for initiating VTE chemoprophylaxis is the progression of intracranial hemorrhage on imaging. The relevant modified Berne–Norwood criteria, a tiered approach guiding VTE chemoprophylaxis initiation in TBI patients, have shown efficacy and safety in VTE prevention and are presented in Table 2 [60,97,98,99]. In the same table, the risk assessment profile score for the risk of VTE prediction is also presented [78]. Knowledge of both scales can assist clinicians in making informed decisions on VTE chemoprophylaxis for their sTBI patients.

The use of IVCFs as a primary VTE prophylactic measure is controversial. Current evidence suggests that this costly and invasive approach should only be considered as a temporary measure before the initiation of prophylactic anticoagulation in patients who cannot be safely anticoagulated within the first 7 days after sTBI. Given that the complications associated with an IVCF increase substantially the longer it remains in situ, all retrievable filters should be removed as soon as prophylactic anticoagulation can be safely started [100]. In rare cases where a trauma patient faces an extremely high risk of complications from VTE chemoprophylaxis over an extended period, the risks and benefits of IVCF placement must be carefully weighed. If IVCF placement is necessary, structured follow-up programs are crucial to resume anticoagulation when safe, increase retrieval rates, and detect complications [60].

The standard approach for diagnosing suspected PE includes assessing pre-test probability, followed by D-dimer testing and then radiological imaging. This approach is not suggested by current guidelines for patients who are hemodynamically unstable and may not be suitable for mechanically ventilated patients [14]. These patients were not included in diagnostic studies and transporting them for computed tomography pulmonary angiography (CTPA) might not be possible. In such cases, echocardiography can identify acute cor pulmonale with PE-related right ventricle (RV) pressure overload and septal dyskinesia. The absence of RV abnormalities on an echocardiogram likely rules out PE as the cause of hemodynamic instability. However, echocardiography does not offer perfect specificity for diagnosing PE, as signs of RV overload or dysfunction may also be found in the absence of acute PE, and may be due to concomitant cardiac or respiratory disease [101]. Furthermore, RV dilation and injury in cardiac arrest patients may be due to the arrest itself rather than its causes. Identifying an accompanying RV thrombus, or deep vein thrombosis through lower limb compression ultrasonography, can help confirm the PE diagnosis in suspected cases [14,80].

Although the majority of hemodynamically stable PE cases can be managed with therapeutic AC [102], this is not enough for the management of high-risk PE [94,103]. In patients without hemodynamic instability, the use of validated scores combining clinical, imaging, and laboratory PE-related prognostic factors may be considered to further stratify the severity of the acute PE episode. Clinical scores that integrate the severity of PE and comorbidities are available, with the Pulmonary Embolism Severity Index (PESI) being the most extensively validated. The key strength of the PESI is its reliable identification of patients at low risk for 30-day mortality (PESI classes I and II). Due to the complexity of the original PESI, which includes 11 differently weighted variables, a simplified version (sPESI) has been developed and validated. The prognostic performance of the sPESI has been confirmed in observational cohort studies [14]. Current guidelines recommend that the assessment of the RV by imaging methods or laboratory biomarkers (e.g., troponin) should be considered, even in the presence of a low sPESI or a negative sPESI.

The management of PE in patients with brain injury involves multiple medical specialties. For patients with sTBI and moderate- or high-risk PE, the clinical situation is particularly complex, necessitating immediate treatment and prompt, challenging clinical decisions. Effective management requires collaboration among emergency physicians, cardiologists, cardiac surgeons, interventional radiologists, angiologists, intensivists, and neurosurgeons [104,105]. Optimal cooperation among these specialties can be achieved by establishing pulmonary embolism response teams (PERTs), organized by each center according to its protocols and needs [106,107]. These teams aim to rapidly assess and treat patients, implement necessary interventions promptly, monitor patient progress, and collect data for clinical studies on this topic [105]. Similar approaches have been successful in managing other life-threatening conditions requiring immediate intervention, such as ischemic and hemorrhagic stroke and acute myocardial infarction [108]. While the impact of PERTs on patient outcomes is still under study, with no conclusive evidence of clear benefits [109], it has been observed that PERT involvement is associated with the application of more advanced treatments, such as catheter-directed thrombectomy and ECMO, and with shorter hospital stays, without increasing bleeding or other adverse complications rates, especially in cases of massive or sub-massive pulmonary embolism [109,110].

## 5. Limitations

We conducted an extensive review of the medical literature from the past five years on TBI and PE; however, our work has several limitations. We only searched two bibliographic sources, PubMed and Scopus, which may have led to the omission of some papers from our review. To minimize this risk, we included relevant papers on each discussed topic in the discussion section by conducting additional separate searches. Moreover, we did not include papers on anticoagulation reversal and the management of patients already on anticoagulation. We chose to exclude papers on this topic because, in our opinion, it warrants a separate review, particularly considering the newer anticoagulants and their antidotes. Finally, regarding the treatment of high-risk PE in sTBI, it is worth mentioning that studies are examining a population with extremely high baseline mortality for a rare complication (PE) that independently carries a high mortality rate, specifically focusing on a rare subtype of high-risk/hemodynamically unstable PE. This significantly reduces the likelihood of detecting a true relationship between PE in sTBI and mortality without an extremely large sample size.

## 6. Areas of Future Research

Several topics relevant to the management of PE in sTBI warrant further research. One such topic is the optimal timing for initiating VTE prophylaxis, which requires results from randomized trials. The timing of PE occurrence to injury time is also poorly discussed, though it is crucial when considering treatment options for severe PE. Furthermore, we identified significant variations in current practices for both VTE prophylaxis and PE treatment. According to our findings, a unified approach to what constitutes early versus late chemoprophylaxis is needed. Additionally, there is limited evidence regarding the use of mechanical thrombectomy. Finally, further research on the pathophysiology of the hypercoagulable state in TBI could, in our opinion, support a better understanding of the disease and assist in the development of personalized management of brain-injured patients [83].

## 7. Conclusions

Despite its importance, the topic of PE in sTBI patients is currently understudied and under-addressed. There is variability among different papers, and consequently, among different investigating centers, regarding the timing, dosing, treatment, and definitions of early versus late VTE prophylaxis and in the management of PE in TBI. Each patient presents a unique challenge to clinicians, and the need for shared decision-making is highlighted in most studies. Additionally, in the era of personalized medicine, little is known about identifying patients who would benefit most from early prophylaxis or more aggressive treatment of concomitant PE.

## Figures and Tables

**Table 2:** Modified Berne–Norwood criteria and risk assessment profile score.

Modified Berne-Norwood Criteria		VTE Risk/RAP Score Points
Consider: IVC filter, lower-extremity duplex screening for DVT	**High risk** ICP monitor in situ CO Evidence of progression at 72 h	High BMI, cancer, coagulation abnormalities, CVC in the femoral vein, RBC transfusion > 4 units, post-operative, chest/head and/or abdomen AIS >2, 40–60 y.o. **2 points**
CTH stable: Initiate pharmacologic prophylaxis at 72 h	**Moderate risk** SDH > 8 mm EDH > 8 mm Cerebral contusion or IVH >2 cm Multiple contusions in a single lobe SAH with abnormal CTA Evidence of progression at 24 h	VTE Hx, major venous repair, spinal fractures, GCS < 8, 60–75 y.o. **3 points**
CTH stable: Initiate pharmacologic prophylaxis at 24 h	**Low risk** No moderate- or high-risk criteria	Spinal cord injury, severe lower extremity and/or pelvic fracture, ≥75 y.o. **4 points**

VTE, venous thromboembolism; CTH, computerized tomography of the head; SDH, subdural hematoma; EDH, epidural hematoma; SAH, subarachnoid hemorrhage; CTA, computerized tomography angiogram; ICP, intracranial pressure; CO, craniotomy; IVC, inferior vena cava; DVT, deep vein thrombosis; RAP, risk assessment profile; BMI, body mass index; Hx, History; CVC, Central venous catheter; RBC, red blood cells; AIS, abbreviated injury score; GCS, Glasgow coma scale; y, years.

## Data Availability

Not applicable.
